# Seeking Meaning: Incorporating Linguistic Information in Cross‐Situational Verb Learning

**DOI:** 10.1111/cogs.70099

**Published:** 2025-08-05

**Authors:** Chi‐hsin Chen, Yayun Zhang, Chen Yu

**Affiliations:** ^1^ Department of Psychology University of Liverpool; ^2^ Language Development Department Max Planck Institute for Psycholinguistics; ^3^ Department of Psychology The University of Texas at Austin

**Keywords:** Cross‐situational learning, Verb learning, Linguistic information, Human Simulation Paradigm, Word‐referent mapping, Word‐meaning mapping

## Abstract

Learning the meaning of a verb is challenging because learners need to resolve two types of ambiguity: (1) word‐referent mapping—finding the correct referent event of a verb, and (2) word‐meaning mapping—inferring the correct meaning of the verb from the referent event (e.g., whether the meaning of an action word is TURNING or TWISTING). The present work examines how adult learners solve this challenge by utilizing both in‐the‐moment linguistic information within individual learning situations and cross‐situational statistical information across multiple learning situations. We investigate how different cues provided in the moment affect information selection and how cross‐situational learning as a general computational mechanism allows for information integration over time. Two experiments were designed based on a Human Simulation Paradigm, in which adult learners were presented with a sequence of short videos from parent−toddler toy play and asked to guess a mystery verb the parent produced in each video. In Experiment 1, we compared individual learning situations containing linguistic information to the exact same learning scenes without linguistic information and found that linguistic information helped learners narrow down the meaning of a verb embedded in individual situations, which was consistent with prior research. In Experiment 2, the videos sharing the same target verb were presented in a blocked design to incorporate cross‐situational statistics for the same verb. We measured the variability, convergence, and accuracy of participants’ guesses. Within‐trial linguistic information allowed learners to quickly narrow down their search space and focus on a few relevant aspects in a scene, while cross‐situational learning allowed them to fine‐tune their learning further across trials. Our findings support a unified account wherein within‐trial linguistic information and cross‐situational statistical information are integrated for more efficient verb learning.

## 1 Introduction

Imagine traveling to a foreign country where you do not speak the local language. You go to a park and see a mother and a child playing with some toy animals. The mother repeats a word while making a part of a toy dog move. What do you think the word means? There are two types of ambiguity in this example scenario. The first type of ambiguity involves finding the referent object or event of the word, subsequently called the word‐referent mapping problem. The word could refer to the mother's action, but also to many other things or events happening in the scene. The second type of ambiguity involves inferring the correct meaning of the word from the target object or event, subsequently called the word‐meaning mapping problem. Let us assume that we know the referent of the word, and it refers to the mother's action. But does it mean TOUCHING THE TOY DOG,^1^ HOLDING, MOVING in general, MOVING A PART OF A TOY (DOG), or MOVING A PART OF A TOY DOG IN A SPECIFIC WAY? A single observation of an action in a visual scene is often insufficient for learners to find out the correct meaning of the word (Gleitman, Cassidy, Nappa, Papafragou, & Trueswell, 2005; Medina, Snedeker, Trueswell, & Gleitman, 2011; Snedeker & Gleitman, 2004). Now imagine that, instead of knowing nothing about the language, you actually know a fair number of words surrounding the novel word. You hear the mother say “Look, I can [UNKNOWN WORD] its tail.” This additional linguistic information allows you to narrow down the word's meaning to a few options, such as MOVE or SHAKE. Even though there is still uncertainty in the exact meaning of the word, you are reasonably confident that your guesses are semantically close to the target word.

The overarching goal of the present work is to investigate how human learners incorporate linguistic information experienced within each learning situation to narrow down the meaning of a word when they encounter the word repeatedly across different learning situations. We will focus on a computational mechanism—cross‐situational learning—that allows within‐trial information to be integrated in finding the word's meaning across time. To closely reflect the input young children receive in their daily interactions, we will use first‐person‐view scenes collected by using a head‐mounted camera worn by toddlers during naturalistic parent−child interactions. Even though our research used adult verb learning as a test case, the general computational principles regarding information selection in the moment and information integration across time can be applied to children's learning and to learning other types of words and even other more general learning problems, such as pattern or rule extraction (Gentner, 1983; Marcus, Fernandes, & Johnson, 2007), structure or sequence learning (Baker, Olson, & Behrmann, 2004; Turk‐Browne & Scholl, 2009; Turk‐Browne, Jungé, & Scholl, 2005), and forming associations between events and objects (Wasserman & Miller, 1997).

In the current word learning literature, how learners use within‐trial information to learn words and how learners gather information across trials to learn words tend to be studied using different paradigms and with different theoretical focuses. For example, studies on how learners use linguistic information in word learning tend to focus on how linguistic information allows learners to go beyond pure observation of a visual scene and helps learners fast‐map a word to its target object or target event (e.g., Gillette, Gleitman, Gleitman, & Lederer, 1999; Nappa, Wesssel, McEldoon, Gleitman, & Trueswell, 2009; Piccin & Waxman, 2007; Snedeker & Gleitman, 2004; Yuan, Fisher, & Snedeker, 2012). On the other hand, studies focusing on how learners gather information across situations tend to focus on how statistical information, such as word‐object co‐occurrences or word‐event co‐occurrences, aggregated across situations helps learning (Akhtar & Montague, 1999; Monaghan, Mattock, Davies, & Smith, 2015; Scott & Fisher, 2012; Yu & Smith, 2007; Yurovsky, Fricker, Yu, & Smith, 2014). Even though some recent studies included linguistic information, such as morphological cues or syntactic information, in statistical learning paradigms, the main focuses tended to be (1) whether or not these cues could be incorporated in statistical word learning and (2) whether and/or how the addition of these cues helped learners solve the *word‐referent mapping* problem (e.g., Y. Chen, LaTourrette, & Trueswell, 2024; Dunn, Frost, & Monaghan, 2024; Finley, 2023; Monaghan et al., 2015, 2024). In this paper, we used multiple measures to examine how within‐trial linguistic information is integrated across learning situations to simultaneously solve the two mapping problems—*word‐referent* and *word‐meaning mapping* problems. Importantly, we employed different measurements and analyses to provide converging evidence on *the real‐time process of how learning unfolded over time*. Our major discovery is that learners utilized within‐trial linguistic information to quickly zoom in on the referent event, and then used aggregated information across learning situations to further fine‐tune their learning. Our findings show that linguistic information presented in each learning situation and cross‐situational information gathered across learning situations allowed learners to address both word‐referent and word‐meaning mapping problems. Linguistic and cross‐situational information worked together to lead to successful verb learning.

### 1.1 Learning verbs from everyday parent−child interactions is hard

Verbs are hard to learn (Gentner, 1982; Gleitman et al., 2005; Piccin & Waxman, 2007; Snedeker & Gleitman, 2004; Swearengin et al., 2025). When we take a young learner's point of view, it becomes clear that verbs are hard to learn for several reasons. First, in daily social interactions, different actions are experienced continuously, and finding the action that parents decide to name can be challenging (Friend & Pace, 2011; Merriman & Tomasello, 1995). For example, when a child hears the word *pick*, the scene in view may be someone reaching for an object, grabbing it, and then picking it up. The event can be segmented in different ways and have different event boundaries. Figuring out the exact action or action sequence that the word *pick* refers to can be an onerous task for a word learner. A second related issue is the transiency of actions compared to objects (Gentner, 1982; Gleitman et al., 2005). Mapping a noun to its object referent in view is often easier because the object will likely continue to exist when the word is heard. However, mapping a verb to its target action is more challenging due to the changing and transient nature of actions seen in daily learning environments (Gentner, 1982; Gentner & Boroditsky, 2001; Gleitman et al., 2005; Merriman & Tomasello, 1995). A heard verb and its denoted action event are often not perfectly temporally coupled. It has been shown that even slight asynchrony between a heard word and its referring object or action can make learning the word‐referent mapping difficult (Gogate, Bahrick, & Watson, 2000; Trueswell et al., 2016). Third, some verbs, such as mental state verbs *think* and *like*, are less concrete or perceptible, and the visual scenes alone are often not informative for learners to learn the meanings (Gleitman et al., 2005; Piccin & Waxman, 2007; Snedeker & Gleitman, 2004). On the other hand, action verbs, such as *run* and *push*, are more concrete and perceptible and easier to learn from different visual scenes. Yet, it is estimated that even these concrete action verbs only co‐occur with their visible target actions or events approximately 50% of the time in children's daily interactions (Tovar‐Perez et al., 2025). These are a few reasons why naturalistic visual scenes in daily life may be less informative for verb learning than the visual scenes created in many experimental studies, which often used concrete action verbs depicting simple movements (e.g., Childers, Cutilletta, Capps, Tovar‐Perez, & Smith, 2023; Monaghan et al., 2015; Scott & Fisher, 2012). Further, most verb learning studies tested learning by showing learners different action events and examined whether learners could correctly identify the action that went with the verb (Y. Chen et al., 2024; Childers et al., 2023; de Carvalho, Dautriche, Fiévet, & Christophe, 2021; He, Kon, & Arunachalam, 2020; Maguire, Hirsh‐Pasek, Golinkoff, & Brandone, 2008; Monaghan et al., 2015; Scott & Fisher, 2012; Yuan et al., 2012; Yuan & Fisher, 2009). Essentially, the main focus was whether learners could solve the *word‐referent mapping problem*. Yet, it is often unclear from those experiments whether learners also solved the *word‐meaning mapping problem*. For example, in an event where someone is running after another person, the verb can be either *chase* or *flee*, depending on the perspective the speaker takes (Gleitman et al., 2005). This type of ambiguity is often not tested in most verb learning studies (except for studies using the Human Simulation Paradigm [HSP] discussed below).

### 1.2 Cross‐situational learning as a statistical solution to word learning

Many studies have shown that adults, children, and even infants are equipped with the ability to accumulate statistical information across learning situations and use the aggregated information to learn novel words (Akhtar & Montague, 1999; Benitez, Yurovsky, & Smith, 2016; C. Chen & Yu, 2022; Childers & Paik, 2009; Dautriche & Chemla, 2014; Hendrickson & Perfors, 2019; Monaghan et al., 2015; Scott & Fisher, 2012; Smith & Yu, 2008; Suanda, Mugwanya, & Namy, 2014; Vlach & Johnson, 2013; Yu & Smith, 2007). Many types of words, including concrete nouns, verbs, adjectives, higher‐level object category labels, and subordinate‐level object names, can be learned through gathering statistical evidence of word‐object or word‐event co‐occurrences across different learning situations (Akhtar & Montague, 1999; C. Chen, Zhang, & Yu, 2018; Childers et al., 2023; Monaghan et al., 2015, 2024; Rebuschat, Monaghan, & Schoetensack, 2021; Scott & Fisher, 2012; Wang & Trueswell, 2019; Yu & Smith, 2007).

The successful demonstration of cross‐situational learning in laboratory experiments raises a valid question about whether this solution can be generalized to word learning in the real world. A comparison between the previous cross‐situational learning experiments designed in the laboratory and word learning in the real world reveals two noticeable distinctions. First, as mentioned previously, to learn a word in the real world, a young learner must resolve two types of ambiguity: word‐referent mapping (e.g., whether a word refers to an action or object) and word‐meaning mapping (e.g., whether the meaning of an action word is MOVING or SHAKING or whether the meaning of an object word is DOG or PET). However, most cross‐situational learning experiments focus only on the word‐referent mapping problem and use easy‐to‐identify objects for noun and adjective learning and/or lab‐created, well‐defined action events for verb learning (e.g., Akhtar & Montague, 1999; Childers et al., 2023; Monaghan et al., 2015; Scott & Fisher, 2012; Yu & Smith, 2007). Even though a few recent studies included designs that tapped into the word‐meaning mapping problem and tested whether learners mapped a novel word to objects at different hierarchical levels (e.g., all dogs or a type of dog, C. Chen et al., 2018; Wang & Trueswell, 2019, 2022). It is unclear whether and how cross‐situational learning addresses the verb‐meaning mapping problem, which is the focus of the present research.

Second, individual learning trials in cross‐situational learning experiments are most often composed of a small number of visual objects and spoken words. Everyday learning contexts seem to be a lot “messier.” In contrast to studies using simplified stimuli or events, several studies using visual scenes collected during parent−child toy play have cast doubts on the utility of cross‐situational learning with more naturalistic stimuli (Gleitman et al., 2005; Piccin & Waxman, 2007; Snedeker & Gleitman, 2004). These studies showed that both adult and child learners failed to use the cross‐situational solution even after seeing the visual scenes containing the referred events several times. For example, using the HSP, Gleitman and colleagues presented adult learners with videos collected during parent−child play with the sound muted and a beep inserted at a time when either a noun or a verb was produced by the parent in the video (Gleitman et al., 2005; Snedeker & Gleitman, 2004). Participants were asked to guess the mystery word that was being produced in each video. The researchers found that even though some videos contained highly informative visual information that allowed learners to successfully guess the mystery word being produced at the moment of the beep, most videos tended to be ambiguous or even uninformative (Gleitman et al., 2005; Medina et al., 2011; Snedeker & Gleitman, 2004). Importantly, verbs often cannot be learned from the visual scenes alone, even after learners have multiple exposures to the visual scenes where a verb is produced (Gillette et al., 1999; Gleitman et al., 2005; Piccin & Waxman, 2007; Snedeker & Gleitman, 2004). The high degree of within‐situation ambiguity from individual learning situations prevents learners from integrating cross‐situational statistics from the learning scenes.

The key rationale behind the HSP is to put adult learners into a learning situation similar to that faced by young children. By doing so, researchers can use adult learning systems to quantify the informativeness of the learning situation and systematically examine the relative impact of different types of information in word learning. Importantly, adult learners can often complete a larger number of test trials, which shed light on real‐time learning; such information is harder to obtain with child participants. This design is similar to the Ideal Observer approach used in vision research, which has led to the generation of testable predictions and hypotheses, the design of new experiments, and the development of explanations and theories (Geisler, 2011; Sims, Jacobs, & Knill, 2012). Despite the in‐principle utility of the HSP, the original HSP does not perfectly serve the purpose because the videos used in the experiments are taken from a third‐person perspective (Gillette et al., 1999; Gleitman et al., 2005; Piccin & Waxman, 2007; Snedeker & Gleitman, 2004). Studies using head‐mounted cameras have shown that third‐person views differ substantially from the egocentric views that young children actually perceive (Smith, Yu, & Pereira, 2011; Yoshida & Smith, 2008; Yu & Smith, 2012; Yurovsky, Smith, & Yu, 2013). The only pertinent view for young children's learning should come from the children's own perspective. In fact, adult participants can learn word‐object mappings better when stimuli are taken from egocentric rather than third‐person‐view videos recorded in the same toy‐play session (Yurovsky et al., 2013; Zhang, Yurovsky, & Yu, 2021). In the case of verb learning, the young children's own perspective limits their perceptual input, thereby constraining the problem of referential ambiguity (Yu & Smith, 2012). Meanwhile, young learners do not hear verbs in isolation. Instead, verbs are often accompanied by other words that they already know. Here, we hypothesize that linguistic information can be particularly useful for resolving the word‐meaning mapping problem when learners encounter learning scenes taken from naturalistic parent−child interactions.

### 1.3 Learning words using linguistic information

In a noisy restaurant, if you hear part of a sentence, “It can [LOUD NOISE] its tail,” you can have a fairly good guess of the missing word based on the linguistic information, which provides both syntactic and semantic cues that greatly constrain the possible words that can occur in an utterance (Gillette et al., 1999; Gleitman et al., 2005; Piccin & Waxman, 2007). Based on the syntax, the mystery word is unlikely to be a noun (e.g., *dog*) or an adjective (e.g., *yellow*). Instead, it is likely to be a verb. Based on the semantics, the mystery word is likely an action performed by something with a tail, and the action involves a tail (e.g., *move*, *wag*, *shake*, or *chase*).

Starting from an early age, children are able to use linguistic, particularly syntactic, information to learn verbs (C. Fisher, Gentner, Scott, & Yuan, 2010, 2020; Gleitman, 1990; He et al., 2020; Naigles, 1996; Naigles & Hoff‐Ginsberg, 1995; Nappa et al., 2009; Yuan et al., 2012). For example, when hearing a sentence “She is gorping her” presented with two scenes, one involving two participants with one woman rotating another woman on a swivel chair and the other scene involving one participant with one woman bouncing on a yoga ball, 21‐ and 19‐month‐old children are more likely to look at the scene with two participants (Yuan et al., 2012). Using syntactic information to help verb learning is a process known as syntactic bootstrapping (Fisher et al., 2010, 2020; Gleitman, 1990; Naigles, 1990; Naigles & Hoff‐Ginsberg, 1995). One fundamental assumption of learning through syntactic bootstrapping is that every single utterance or sentence only contains partial distributional information about how a verb is used (Fisher, Jin, & Scott, 2020; Lidz, 2020; Naigles, 1996; Naigles & Hoff‐Ginsberg, 1995). Therefore, to successfully learn a verb's meaning via syntactic bootstrapping, learners need multiple encounters with a verb to learn how it can be used in different contexts with different syntactic environments. The core idea is that learners need to gather information across various learning situations to acquire the meaning of a verb. From this perspective, cross‐situational learning is the fundamental operating mechanism that enables learners to acquire a verb's meaning through syntactic bootstrapping. Yet, the role of cross‐situational learning is often either ignored entirely or only lightly touched upon in previous experimental studies focusing on how learners use linguistic information in their verb learning, despite the fact that those studies often examine learning outcomes after participants have gathered information across different learning trials, which in essence is a cross‐situational learning manipulation (Gillette et al., 1999; Gleitman et al., 2005; Snedeker & Gleitman, 2004). Our present work focuses on cross‐situational learning as the computational framework that operates across multiple learning situations, allowing learners to incorporate different types of within‐trial information—be it visual, linguistic, or other types of information—across multiple situations to learn the meanings of novel words.

### 1.4 The current research

In this research, we used the HSP, in which adult learners were presented with short egocentric videos taken from parent−toddler toy play and had to guess a mystery verb the parent produced at a given moment in the video (Gillette et al., 1999; Gleitman et al., 2005; Piccin & Waxman, 2007; Snedeker & Gleitman, 2004). We picked videos with concrete action verbs, as they are the first verbs learned by young children^2^ (Frank, Braginsky, Yurovsky, & Marchman, 2017; Gillette et al., 1999; Snedeker & Gleitman, 2004). We used concrete action verbs as the test case to investigate how different types of information affect learners’ information selection within individual situations and information integration across multiple situations.

Even though the HSP has been criticized for being very different from children's first language learning (for discussions of the advantages and limitations of the HSP, see Gleitman et al., 2005; Kako, 2005; Snedeker & Gleitman, 2004), this method resembles second language learning scenarios and allows us to use adult learning systems to quantify and systematically examine the informativeness of different learning situations for child word learning. The HSP also enables us to test learners repeatedly and provides high‐density data in the course of cross‐situational learning, which is often harder to obtain with young children. Later in the Discussion, we will return to this topic. Another reason to use this paradigm is that several previous studies using this paradigm have shown that verb learning often does not benefit from cross‐situational encounters of visual scenes (Gillette et al., 1999; Gleitman et al., 2005; Snedeker & Gleitman, 2004). Therefore, we would like to use the same paradigm for comparison. However, unlike previous studies using the HSP, which often used videos taken from a third‐person view, we used toddlers’ first‐person‐view videos collected by a head‐mounted camera placed on children's foreheads during parent−child play. Compared to the original HSP studies using third‐person view videos, the use of first‐person view videos should better serve the HSP's fundamental purpose.

We conducted two experiments. Experiment 1 used a design without the cross‐situational learning component to establish baselines for comparisons with Experiment 2. We included two conditions in Experiment 1—one containing only visual information (subsequently termed Visual‐only condition) and the other containing both visual and linguistic information (subsequently termed Visual‐and‐linguistic condition). These two conditions allowed us to directly test the effect of added linguistic information on verb learning when learners encountered videos depicting different action events. In each condition, participants were presented with 66 videos extracted from parent−child play and had to guess a target verb produced by the parent in each video. Across the 66 videos, there were 11 different verbs, each presented in six different videos. It is noteworthy that the videos for the same target verb never occurred in two consecutive trials, and participants were never told that some videos may contain the same verb. This design made it challenging for learners to aggregate information across different trials.

In Experiment 2, we tested the role of cross‐situational learning when learners were presented with the same type of information as shown in Experiment 1. But instead of presenting the videos of the same target verb interspersed with the videos for other verbs, all six videos of the same verb were presented in a block. This blocked design allowed participants to aggregate information across trials and use the cross‐situational information to refine their guesses across trials. Similarly, there were two conditions, Visual‐CSL (CSL stands for cross‐situational learning) and Visual‐and‐linguistic‐CSL conditions. The Visual‐CSL condition contained the same stimuli as the Visual‐only condition in Experiment 1, while the Visual‐and‐linguistic‐CSL condition contained the same stimuli as the Visual‐and‐linguistic condition. These two experiments allowed us to systematically test the utility of the cross‐situational learning mechanism in verb learning with naturalistic parent−child play scenes.

In both experiments, we examined the distribution and accuracy of participants’ guesses by using three measures—(1) variability: the number of unique guesses provided by all participants for each video, (2) convergence: the proportion of guesses that belonged to the top choice for each video, and (3) accuracy: the proportion of guesses that matched the verbs produced by parents in the videos.

## 2 Experiment 1

This experiment had two conditions: Visual‐only and Visual‐and‐linguistic conditions. Participants’ task was to use the information provided in individual learning trials to guess the mystery verbs produced by parents.

### 2.1 Methods

#### 2.1.1 Participants

The main goals of Experiment 1 were to test how informative the visual‐only and visual‐and‐linguistic stimuli were and to establish the baselines for comparisons with Experiment 2. Following previous studies, we aimed to recruit a minimum of 36 participants per condition to establish informative baselines (Medina et al., 2011).^3^ Due to the Covid‐19 pandemic, participant recruitment had two phases—in‐person and online. Participants recruited for the Visual‐only condition were tested in‐person in a laboratory setting. Participants recruited for the Visual‐and‐linguistic condition were recruited online using Amazon Mechanical Turk. Because the two groups of participants were recruited using different mechanisms and run in different settings, we recruited another group of 40 participants online to replicate the findings from the Visual‐only condition during the review process of this paper. The findings from this new group of control participants were similar to our original in‐lab study, and the conclusions were the same. In the following, in addition to the descriptive and inferential statistics of our original in‐lab study, we will also report the descriptive statistics from this control group for comparisons.

Recruitment and experimental procedure were approved in advance by the Institutional Review Board at the university where the research was conducted. A total of 122 adults participated in our original study, and 40 in the control condition. All participants gave informed consent prior to participation. Some participants completed fewer than 50% of trials and were excluded from analyses (for details, see below).

Participants in the Visual‐only condition were 52 undergraduate students recruited at a university in the mid‐western United States. After excluding participants who completed fewer than 50% of trials, the final sample contained 45 participants (30 females, mean age: 19.22 years, *SD* = 0.94). For the control group, a total of 40 participants were recruited online. One of them finished fewer than 50% of trials and, therefore, was not included in the analyses. The final sample included 39 participants (25 females, mean age: 40.54 years, *SD* = 13.59; Education level: 13% high school, 5% associate, 23% some college, 44% bachelor's degree, 15% graduate degree).

Participants in the Visual‐and‐linguistic condition were recruited online. A total of 70 participants were recruited. Some of them finished fewer than 50% of trials and, therefore, were not included in the analyses. The final sample included 60 participants (27 females, mean age: 40.17 years, *SD* = 9.83; Education level: 3% never finished high school, 3% high school, 7% associate, 22% some college, 38% bachelor's degree, 17% graduate degree, 10% N/A).

#### 2.1.2 Stimuli

##### 2.1.2.1 Video corpus

**Fig. 1:**
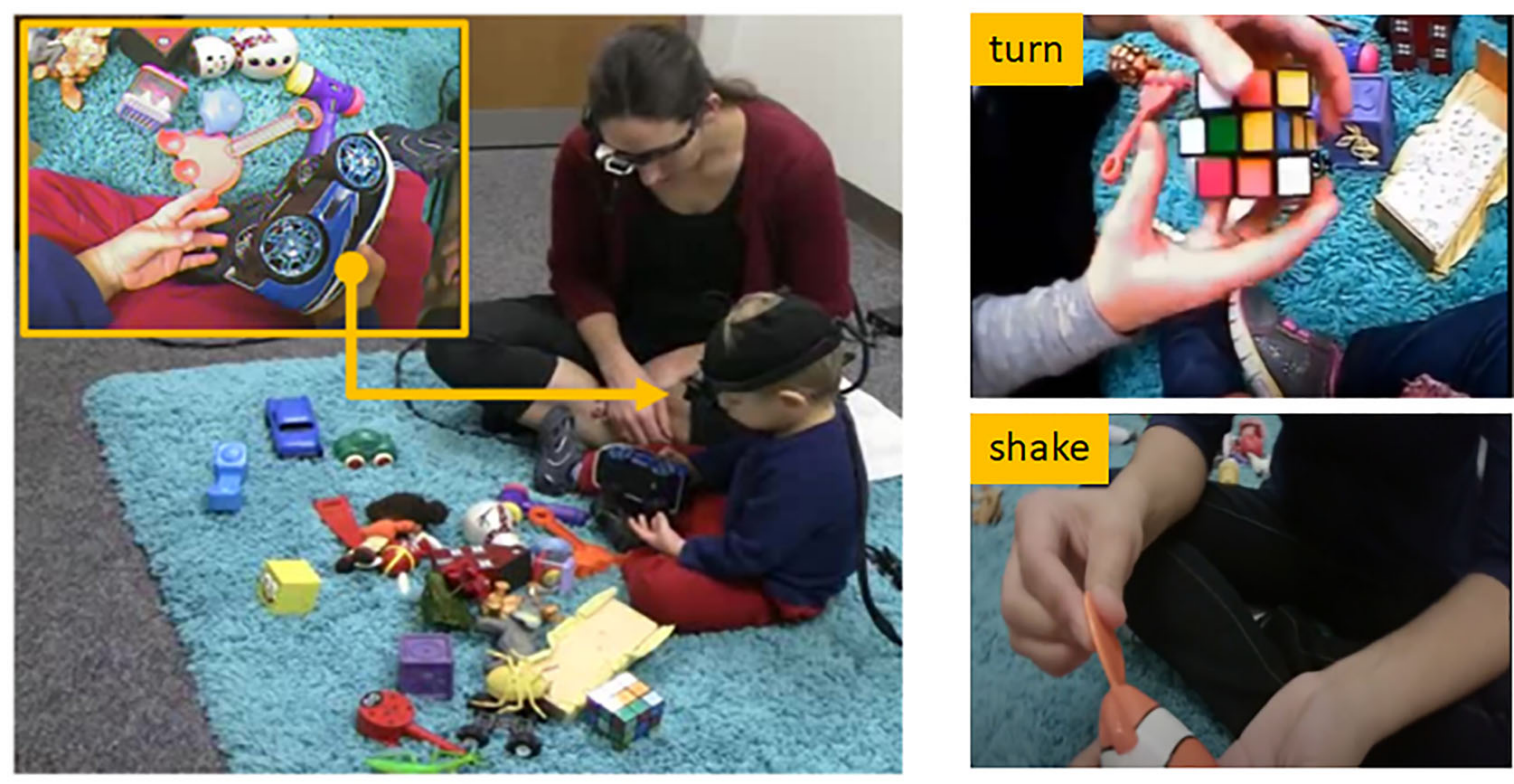
Experimental setup for the parent−child play video corpus. *Note*. The parent and child played with a set of toys in a naturalistic setting. The child's first‐person view was recorded by a head‐mounted camera (top left). We used the videos taken from children's first‐person view in the experiment. The screenshots of two verb instances, *turn* and *shake*, are shown on the right.

The videos were extracted from a corpus of play session recordings from 32 parent−child dyads. The children were between 12 and 25 months of age (19 females, mean age: 19.07 months, *SD* = 3.4). During the play session, the participants were provided with 24 toys (e.g., animals, cars, tools, dolls, and blocks) and instructed to play as they naturally would at home. Each dyad played with the toys for 10 min. During the play session, the parent and toddler both wore a head‐mounted camera that recorded their first‐person view (see Fig. 1). In the current research, we only used the videos recorded from the child's first‐person view.

Parents’ speech recorded during the play sessions was transcribed. A total of 152 unique verbs with 1869 verb instances (ranging from 1 to 168 instances for each verb) were found in the corpus. Based on the transcriptions, we extracted the video streams and created 5‐s video vignettes, each containing a target verb occurring at the third second after the video onset. We then used the following criteria to select the video vignettes to be used in the experiments: (1) the target verb was a concrete action verb (e.g., *walk*, *grab*, *jump*); (2) each target verb had at least 12 video instances in the database; (3) each target verb had video instances coming from at least three different parent−child dyads; and (4) each target verb had videos with the verb actions performed by or directed toward at least three different objects across different videos. After applying these criteria, a total of 11 verbs were selected: *shake*, *hold*, *eat*, *fall*, *drive*, *turn*, *put*, *cut*, *fit*, *knock*, and *stack*.

We selected six video vignettes for each verb (6 videos * 11 verbs = 66 videos) and made sure that the videos for each verb came from at least three different dyads^4^ and the verbs were performed by or acted on at least three different objects across videos. These videos were then used in both Visual‐only and Visual‐and‐linguistic conditions. Four additional videos with verbs not used in the experiments (e.g., *press* and *throw*) were selected as practice trials used in each condition.

##### 2.1.2.2 Visual‐only videos

In the Visual‐only condition, the original audio was muted, and a beep was inserted at the onset when the parent produced the target verb. The video vignettes were cut so that the beep in a video occurred at the beginning of the third second after the onset of the video clip.

##### 2.1.2.3 Visual‐and‐linguistic videos

The video clips were the same as the ones in the Visual‐only condition. We went back to the speech transcripts and extracted the utterances where the verbs occurred. The utterance boundaries were defined by pauses lasting at least 400 ms (Suanda, Smith, & Yu, 2016; Suarez‐Rivera, Smith, & Yu, 2019; Yu & Smith, 2012). We added the whole utterance into each video, but with the target verb replaced by a beep sound. To make sure that the utterances did not contain additional speaker, emotional, or prosodic information, we used Amazon Polly's text‐to‐speech natural speech synthesizer (with Salli's voice) to generate the utterances for all videos in this condition. Example videos can be seen at the Open Science Framework (OSF) page [https://osf.io/3dfzc/?view_only=5d8dc779646e495b898b908282c122e9].

With the target verbs included, the original utterances contained between 1 and 13 words (mean = 5.52, *SD* = 2.54). After replacing the target verbs with beeps, the linguistic frames (i.e., linguistic information surrounding the beeps), on average, contained 4.47 words (*SD* = 2.54). There are two things worth mentioning about the utterances used in this condition. First, four of the 66 utterances (6%) extracted from the transcripts were single‐word utterances—each utterance contained only one verb (each containing “shake,” “drive,” “cut,” or “stack”). One additional utterance contained a verb followed by the word “yes” (“Cut, yes”). Technically, these five utterances did not provide *additional* linguistic information when the verbs were replaced by a beep sound. However, because the data reflected the characteristics of the input young children received in naturalistic play interactions, which often contained very simple or incomplete sentences, we did not modify the utterances to artificially add in more linguistic information. If the stimuli we used in the current Visual‐and‐linguistic condition can help learners guess what verbs parents produced in the videos, we predict that the facilitative effect would be even stronger with a “cleaner” design where utterances are composed of complete sentences with more informative linguistic information. Second, in two utterances extracted from the transcripts, the target verb occurred more than once (“*Knock*, *knock*, *knock*, anybody home?” and “Reach, okay, *stack*, *stack*.”). In these two utterances, each target verb occurrence was replaced by a beep to reflect the characteristics of the linguistic structure in natural speech. As a result, one utterance had three beep sounds and the other had two. The first beep in each video occurred at the beginning of the third second after the video started.

For the rest of the utterances (after excluding the single‐verb utterances and utterances with multiple target instances), five had the target verb (and, therefore, the replacing beep) occurring at the beginning of the utterance^5^ (e.g., “*Shake* it!”), 42 in the middle of the utterance (e.g., “It can *shake* its tail.”), and 12 at the end of the utterance (e.g., “See if this one can *shake*.”).

#### 2.1.3 Procedure

The experiment took approximately 20 min to finish. Participants were instructed to carefully watch short videos from parent−child play sessions and pay attention to the moments of the beeps in the videos when the parents produced concrete action verbs. They were asked to guess what verb the parent produced in each trial and type in the answer using a correctly spelled verb in the present tense. Participants were never told that some videos may contain the same verb.

Two lists with different trial orders were used. The order of the video clips in each list was assigned pseudo‐randomly with the constraint that the videos of the same target verb did not occur in two consecutive trials (lag range: 5–22). In each list, each video was played only once. After watching each video, participants had up to 40 s to enter their answers. After providing an answer, they then pressed a button to proceed to the next trial. No feedback was provided.

An answer was coded as correct if it matched the verb the parent produced in the video. Tenses were corrected prior to coding so that all answers were presented in the present tense (e.g., *turned* or *turning* corrected to *turn*).

### 2.2 Results

In the following, we test whether the information provided in the videos was sufficient for participants to guess the verbs produced by parents and whether participants’ guesses converged and were correct. We had three measures: (1) the variability of guesses, as measured by using the number of unique guesses all participants provided for the target verb in each video; (2) the convergence of guesses, as measured by using the proportion of guesses belonging to the top choice for each video; and (3) the accuracy of guesses, as measured by using the proportion of guesses that matched the target verbs parents produced in the videos. The unique guesses and top choice analyses were conducted using linear mixed‐effects models from the *lme*4 package in R studio. The accuracy‐related analyses were conducted using generalized linear mixed‐effects models, because the accuracy of a guess was scored as 1 (correct) or 0 (incorrect) for each trial. In the following analyses, we will compare the performance between different conditions. We predict that the additional linguistic information in the Visual‐and‐linguistic condition will allow learners to converge on their guesses and increase their accuracy. The data and analyses can be found on the Open Science Framework (OSF) site [https://osf.io/b7mjz/?view_only = 1d06291e5f814016804525175a187559].

#### 2.2.1 Variability of guesses: The number of unique guesses

**Fig. 2:**
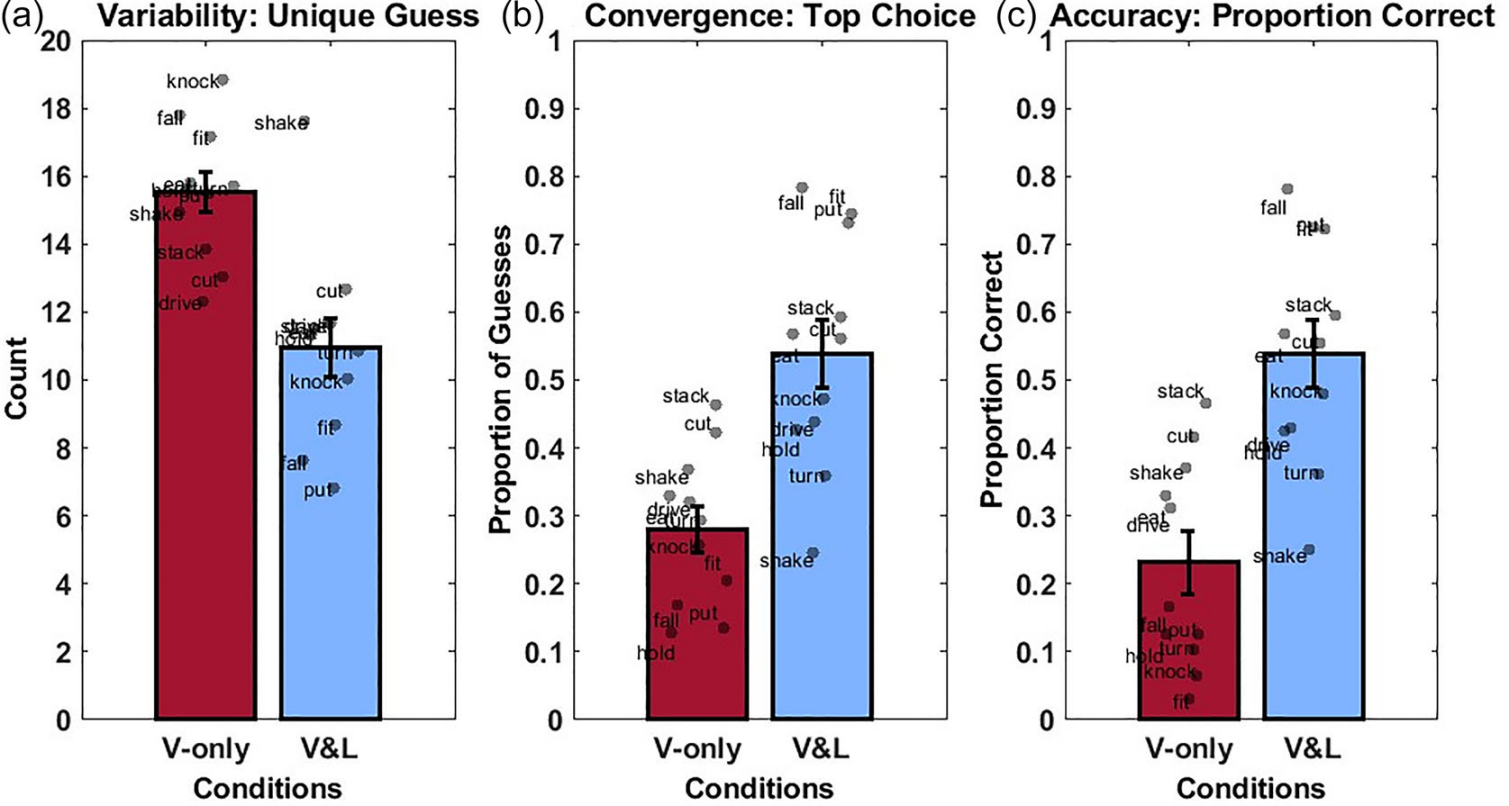
Results in Experiment 1. *Note*. (a) Variability: Number of unique guesses (averaged across verbs) and SE in the Visual‐only and Visual‐and‐linguistic conditions. (b) Convergence: Proportion of guesses belonging to the top choice (averaged across verbs) and SE in the two conditions. (c) Accuracy: Proportion of guesses that were correct (averaged across participants) and SE in the two conditions.

We first report the average number of unique guesses provided by participants in each condition. As shown in Fig. 2a, the 45 participants, as a group, generated an average of 15.52 guesses (*SD* = 3.32) in each verb encounter in the original Visual‐only condition. The 39 control group participants recruited online generated an average of 12.85 verbs (*SD* = 3.49). That is, one unique guess generated by 3.03 participants. This is similar to the in‐lab Visual‐only condition, where one unique guess was generated by 2.90 participants. In contrast, the 60 participants in the Visual‐and‐linguistic condition on average generated 10.95 guesses (*SD* = 4.08) in total, with one unique verb generated by 5.48 participants. Next, we tested the effect of different conditions by using a linear mixed model, with the condition (Visual‐only vs. Visual‐and‐linguistic) as a fixed factor, different verbs as a random factor, and the number of guesses as the dependent measure. Participants produced significantly fewer unique guesses in the Visual‐and‐linguistic condition than the Visual‐only condition, β = −4.56, *SE* = 0.63, *t*(120) = −7.22, *p* < .001. It is noteworthy that there were more participants in the Visual‐and‐linguistic condition than in the Visual‐only condition (60 vs. 45). More people, in principle, can provide more unique guesses. However, we found that, even with more participants, the group in the Visual‐and‐linguistic condition had fewer unique guesses. Therefore, the results suggest that additional linguistic information helped participants narrow down their guesses and reduced the guess variability.

#### 2.2.2 Convergence of guesses: The proportion of guesses belonging to the top choice

The second set of analyses focused on the convergence of guesses. We calculated the proportion of all responses that belonged to the top choice for each target verb in each video.

As shown in Fig. 2b, on average, 0.28 (*SD* = 0.11) of all responses corresponded to the top choice in the Visual‐only condition. For the control group recruited online, the mean was 0.31 (*SD* = 0.15). The mean for the Visual‐and‐linguistic condition was 0.54 (*SD* = 0.17). That is, while slightly over one‐fourth of all guesses in the Visual‐only condition belonged to the top choice, over half of all guesses in the Visual‐and‐linguistic condition belonged to the top choice. We then used a linear mixed‐effects model with the condition as the predictor, the individual verbs as a random factor, and the proportion of responses that belonged to the top choice as the dependent variable. Overall, a significantly higher proportion of responses belonged to the top choice in the Visual‐and‐linguistic condition than in the Visual‐only condition, β = 0.26, *SE* = 0.06, *t*(20) = 4.28, *p* < .001. This finding suggests that the addition of linguistic information facilitated the convergence of responses to the top choice.

#### 2.2.3 Accuracy: The proportion of guesses that matched the verbs produced by parents

The last set of analyses focused on the accuracy of guesses (Fig. 2c). We asked whether the accuracies differed as a function of condition by using the condition as a predictor and different participants and different target verbs as random factors.

The proportion of correct answers in the Visual‐only condition was 0.23 (*SD* = 0.42). For the control group recruited online, the mean accuracy was 0.27 (*SD* = 0.19). And the proportion of correct answers in the Visual‐and‐linguistic condition was about twice as high (mean = 0.54, *SD* = 0.50). Between‐group analyses confirmed that the participants in the Visual‐and‐linguistic condition were more accurate in their guesses, β = 1.45, *SE* = 0.08, *z* = 17.69, *p* < .001, *n* of observations = 6930. This finding suggests that adding the linguistic information significantly helped participants guess the verbs parents produced in the videos.

### 2.3 Discussion

In this experiment, we tested whether participants could use the information they received in each video to correctly identify the verbs produced by parents. Consistent with previous findings, when learners only had visual information, their guesses tended to be more variable and less accurate (Gleitman et al., 2005; Snedeker & Gleitman, 2004). Adding linguistic information helped learners narrow down their guesses. Participants as a group generated fewer and more similar guesses in the Visual‐and‐linguistic condition. Furthermore, their guesses were more accurate in that condition. These findings suggest that adding linguistic information helped learners guess the verbs used by the parent in each video.

In this study, the videos sharing the same target verb never occurred in two consecutive trials. In the next experiment, we presented the videos of the same verb in a blocked design to encourage participants to use the information gathered across trials to infer the meanings of the target verbs.

## 3 Experiment 2

In Experiment 2, we investigated how cross‐situational learning allowed learners to fine‐tune their guesses as they gathered information across trials. We used the same stimuli as those in Experiment 1. However, the videos for the same target verb were presented in a blocked design. The close temporal presentation of the videos for the same verb allowed learners to use the information aggregated across trials to refine their guesses. We used the same measures—variability, convergence, and accuracy of guesses—as the dependent variables. But before presenting the findings using these measures in the Results section, we will start by presenting preliminary analyses using an exploratory method, Sankey diagrams, to illustrate the dynamics of participants’ guesses as they aggregate information across learning trials. We will provide more information on this method in the Results section. But the take‐home message from the exploratory Sankey diagrams is that within‐trial linguistic information allowed learners to quickly narrow down their search space, and cross‐situational learning allowed learners to fine‐tune their guesses across trials.

### 3.1 Methods

#### 3.1.1 Participants

Because there is not a single standard way to calculate the effect size for mixed‐effects models, we calculated Cohen's *d* for the accuracy data collected from the two conditions in Experiment 1 as a heuristic. The effect size obtained from Experiment 1 was 0.67. To have .8 power to detect the effect size, the sample size needed per group is 36.

A total of 80 adults recruited from Amazon Mechanical Turk participated. Eight of them completed fewer than 50% of the trials and were excluded from analyses. The final sample contained 72 participants (38 females, mean age: 43.85 years, *SD* = 10.07; Education level: 7% high school, 6% associate, 25% some college, 44% bachelor's degree, 18% graduate degree), with 36 participants in the Visual‐CSL condition and 36 in the Visual‐and‐linguistic‐CSL condition.

#### 3.1.2 Stimuli

The video clips were the same as those used in Experiment 1. Each verb had six video instances, with a total of 66 videos for the 11 verbs used in the studies. However, instead of being presented in an interspersed manner, the videos with the same target verb were presented in the same block. Each block contained six trials. Two experimental lists, each with a different block order and trial order, were used in each condition to ensure that any effects found were not due to any peculiarity of a single list.

#### 3.1.3 Procedure

Participants were informed that they would watch blocks of videos extracted from parent−child play sessions. They were asked to guess the concrete action verb that the parent produced when a beep occurred in a video. They were explicitly informed that all the trials in the same block had the same target verb. They were also told that they could change their answers as they went along or enter the same answer if they believed that the previous guess was correct. However, they were unable to return to a previous trial and change an answer that had already been submitted. After each block, a prompt notified participants that the subsequent trial belonged to a new block with a new target verb. No feedback was given during the experiment.

### 3.2 Results

The measures of interest are: (1) variability—the number of unique guesses; (2) convergence—the proportion of responses belonging to the top choice; and (3) accuracy—the proportion of participants’ responses that matched the verbs produced by parents. In each set of analyses, we will start by first examining whether or not increased encounters with the videos of the same verb helped learners’ guesses in each condition, and then test whether participants in the two conditions in Experiment 2 performed differently. After that, we will compare the findings from Experiment 2 to those in Experiment 1 to provide further evidence of the gain from cross‐situational learning. However, before presenting the measures of interest, we will first present preliminary analyses using Sankey diagrams to illustrate in detail the flow of guesses under different conditions and discuss how different types of information influence the flows.

#### 3.2.1 How did guesses change from one encounter to another?

**Fig. 3:**
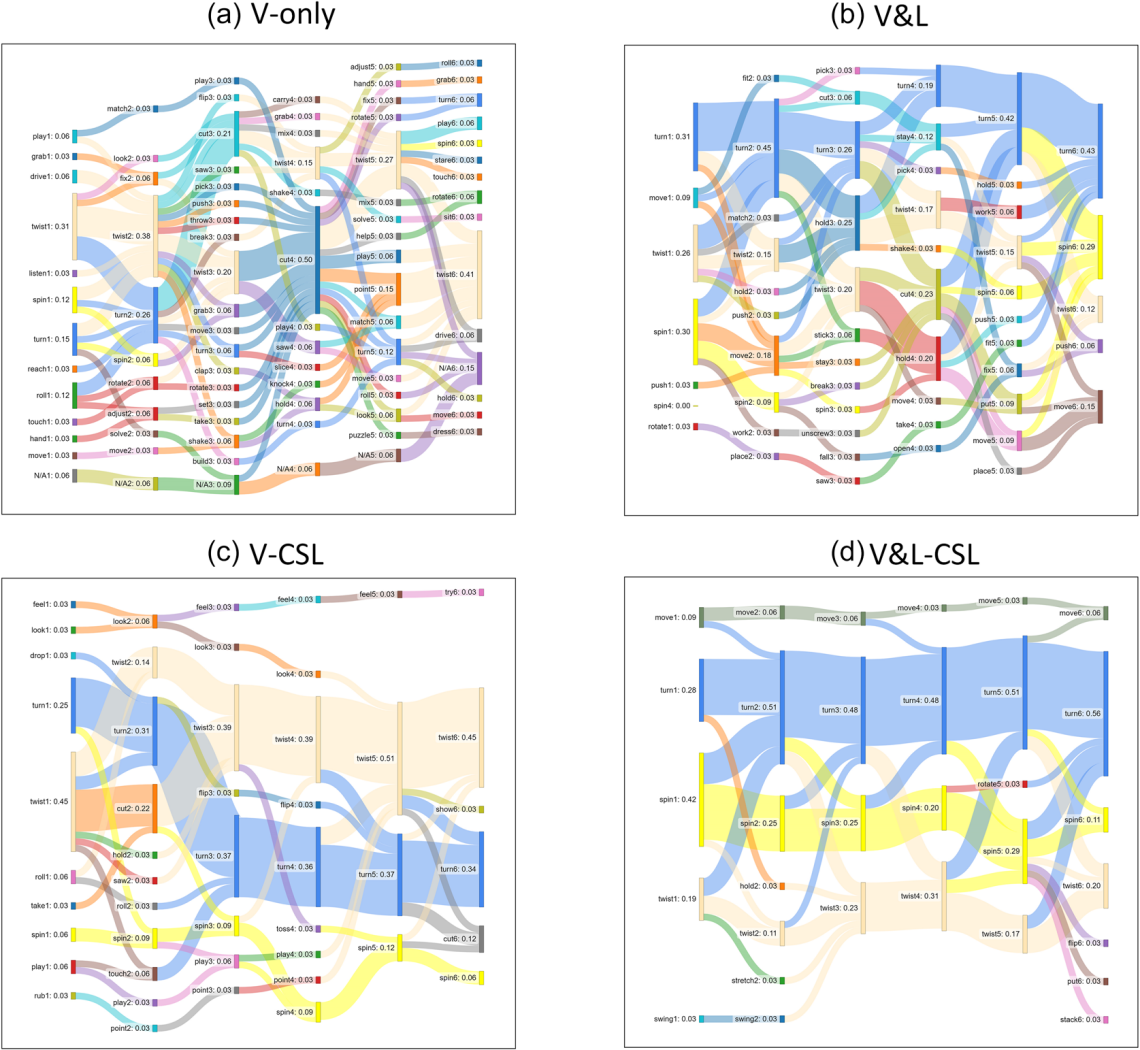
Sankey diagrams representing changes of guesses in different encounters for the verb turn. *Note*. Changes of guesses for the target verb *turn* in the (a) Visual‐only condition in Experiment 1, (b) Visual‐and‐linguistic condition in Experiment 1, (c) Visual‐CSL condition in Experiment 2, and (d) Visual‐and‐linguistic‐CSL condition in Experiment 2. The target verb *turn* was represented in blue nodes and flows. N/A was used when a participant did not provide a guess or when a guess was not a verb (e.g., *dunno*).

To illustrate how aggregating information across learning trials allows learners to fine‐tune their guesses, we will report exploratory analyses that represent the flow of guesses in Experiments 1 and 2 by using Sankey diagrams generated using SankeyMATIC (https://sankeymatic.com/). A Sankey diagram is a visualization tool traditionally used to represent energy flows and their distributions in different states (Schmidt, 2008a, 2008b). We will use this method to depict how participants’ guesses changed (or not changed) from one encounter to another, with the size of nodes and the thickness of each flow indicating the proportion of participants’ guesses belonging to each response verb (Fig. 3). To make different conditions comparable, we used the answers of 36 participants per condition in this set of analyses. For the Visual‐only and Visual‐and‐linguistic conditions from Experiment 1, we included the answers of the first 36 participants. Even though the Sankey diagrams allow for detailed examination of the types and proportions of different guesses in each encounter, our purpose here is to provide a high‐level “big picture” of the general patterns of flow reduction (or nonreduction) across encounters in different conditions. To observe general patterns, it is not necessary to zoom in on each individual node or flow and focus on the nuanced details. However, for readers interested in the detailed patterns, a high‐resolution figure can be found on our OSF site (https://osf.io/6wume/?view_only = c603ce0ccc2c42ef82a6a366dc410b82).

We will present the Sankey diagrams using the target verb, *turn*, as an example. This verb had the lowest overall accuracy across all trials in the Visual‐and‐linguistic‐CSL condition, the condition where participants had the highest overall accuracy among the four conditions in Experiments 1 and 2 (for details, see the Accuracy section below). Across the six trials in the Visual‐and‐linguistic‐CSL condition, the verb *turn* had an overall accuracy of 0.46, which was much lower than the top three verbs with the highest accuracies (*fall*: 0.91, *put*: 0.89, and *fit*: 0.86) in the same condition. The reason why we picked the verb with the lowest accuracy in the Visual‐and‐linguistic‐CSL condition was that we wanted to use it as a *lower‐bound estimate* to show how guesses may converge across trials in verb learning. The patterns likely reflect a worst‐case learning scenario and may be closer to how young children—who likely have less capacity to utilize visual and linguistic information across various learning situations—may perform when they receive these different types of information.

Fig. 3 provides a visualization of participants’ guesses across the six video encounters with the target verb *turn* in each condition. The label for each node (e.g., turn1: 0.31 or spin6: 0.29) has three components: (1) participants’ response verb (e.g., *turn* or *spin*); (2) which encounter the trial represents (1−6 indicating the six encounters); and (3) proportion of guesses belonging to each response verb. For example, turn1: 0.31 indicates that 31% of all guesses were *turn* in the first encounter. And spin6: 0.29 indicates that 29% of all guesses were *spin* in the sixth encounter. The target verb *turn* was represented in blue nodes and flows. The thickness of the blue nodes and flows indicates the proportion of participants who correctly identified the verb in each encounter. It is important to note that, since we used two lists with different trial orders in each condition, the guesses for each encounter illustrated in the diagrams represent the guesses for two distinct videos and may, therefore, contain guesses that differ significantly from one another. However, the changes across different encounters still provide important insights about how different sources of information play a role in verb learning across encounters.

These different diagrams show that when the only information came from the visual scenes (Fig. 3a), learners as a group provided a wide range of guesses. This is likely because they attended to different aspects of the same scenes (e.g., *play*, *roll*, *reach*, *move*, or *turn*) and/or interpreted the salient action(s) in different ways (e.g., *twist*, *turn*, *spin*, *rotate*). Adding linguistic information to the visual scenes (Fig. 3b) greatly helped learners narrow down the search space and reduce the variability of guesses. However, because the videos for the same target verb were not presented in close temporal succession, learners did not benefit much from increased encounters with the videos of the same verb. They continued to provide different guesses across trials based on the visual and linguistic information encountered in each individual video. On the other hand, when the visual scenes were presented in a blocked design (Fig. 3c), learners gradually converged to fewer and fewer verbs across trials. But because the main information source was the visual scenes, learners split between two verbs depicting similar actions, *twist* and *turn*. Both made sense given the visual information alone. When visual and linguistic information was both available and presented in close succession (Fig. 3d), learners used the linguistic information to quickly narrow down the search space from the beginning and gradually converged to the target verb across trials. In the last two encounters, over 50% of participants agreed on the verb *turn*. These diagrams suggest that even though linguistic information had an immediate effect and helped learners narrow down their search space from the first encounter, learners used the information gathered across trials to fine‐tune their learning. As mentioned before, we used *turn* to show the lower bound estimate of the pattern, because it had the lowest overall accuracy in the Visual‐and‐linguistic‐CSL condition. For verbs with higher accuracies (e.g., *fall*: 0.91, *put*: 0.89, and *fit*: 0.86), the majority of guesses converged to the target verb with fewer encounters.

#### 3.2.2 Variability of guesses: The number of unique guesses

**Fig. 4:**
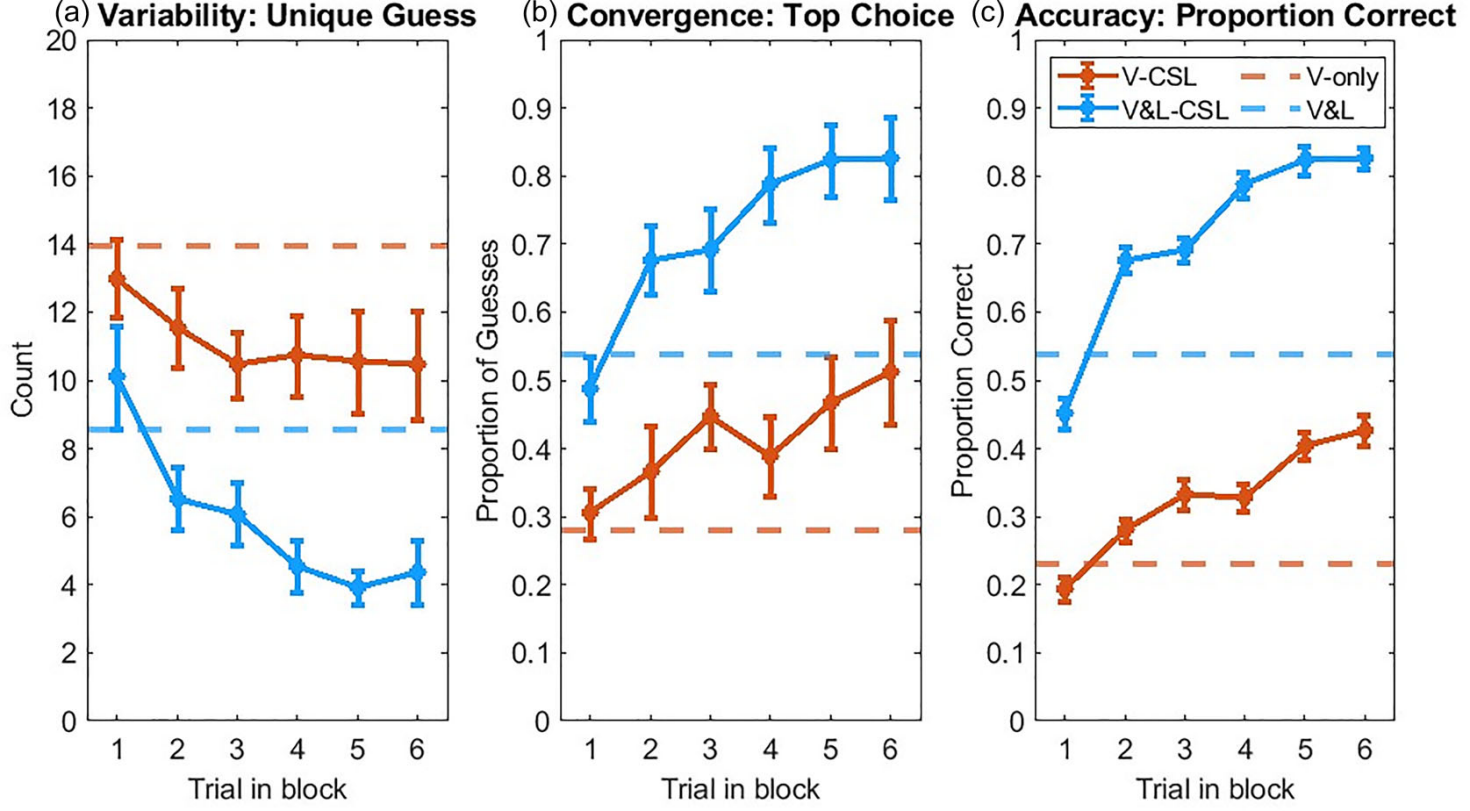
Results in Experiments 1 and 2. *Note*. (a) Variability: Number of unique guesses (averaged across verbs) and SE in the Visual‐CSL, and Visual‐and‐linguistic‐CSL conditions. The dashed lines represent the results taken from the first 36 participants in the Visual‐only and Visual‐and‐linguistic conditions from Experiment 1. (b) Convergence: Proportion of guesses belonging to the top choice (averaged across verbs) and SE in the Visual‐CSL and Visual‐and‐linguistic‐CSL conditions. The dashed lines represent the results taken from the Visual‐only and Visual‐and‐linguistic conditions. (c) Accuracy: Proportion of guesses that were correct (averaged across participants) and SE in the Visual‐CSL and Visual‐and‐linguistic‐CSL conditions. The dashed lines represent the results taken from the Visual‐only and Visual‐and‐linguistic conditions.

The mean number of unique guesses averaged across all six encounters in the Visual‐CSL condition was 11.12 (*SD* = 4.20). As Fig. 4a shows, with increased exposure, the average number of unique guesses decreased from 13 to 10.46. We tested the effect of increased encounters on the number of unique guesses by using a linear mixed effects model with the different verbs as a random factor. With increased encounters, the number of unique guesses reduced across trials, β = −0.44, *SE* = 0.18, *t*(54) = −2.45, *p* < .02. The mean number of unique guesses averaged across all six encounters in the Visual‐and‐linguistic‐CSL condition was 5.92 (*SD* = 3.76). The number decreased from 10.09 to 4.36 between the first and last encounters. Again, we used a linear mixed‐effects model to test the effect of increased encounters on the number of unique guesses and found a significant effect, β = −1.09, *SE* = 0.19, *t*(54) *=* −5.88, *p* < .001. These findings suggest that learners, as a group, gradually reduced their guess variability across encounters in both conditions.

We then conducted linear mixed‐effects models with condition (Visual‐CLS vs. Visual‐and‐linguistic‐CSL) and the number of encounters as fixed effects, the different verbs as a random factor, and the number of unique guesses as the dependent variable. Participants in the Visual‐and‐linguistic‐CSL condition produced significantly fewer unique guesses than their counterparts in the Visual‐CSL condition, β = −2.93, *SE* = 1.45, *t*(118) = −2.02, *p* = .046. Even though the parameter estimate suggests that the number of guesses decreased as the number of encounters increased, the pattern did not reach statistical significance, β = −0.44, *SE* = 0.26, *t*(118) *=* −1.67, *p* = .097. There was no significant interaction between the two fixed factors, β = −0.65, *SE* = 0.37, *t*(118) = −1.73, *p* = .086. These results showed that the additional linguistic information allowed learners to narrow down their search space and had fewer guesses.

##### 3.2.2.1 Between‐experiment comparisons

We further tested the gain from cross‐situational learning by comparing the results between Experiments 1 and 2 (Fig. 4a). Since more participants can potentially produce a larger number of unique guesses, we matched the number of participants in Experiments 1 and 2. To do so, we took a subset of data from Experiment 1 (i.e., the first 36 participants in each condition) and calculated the number of unique guesses for different verbs based on the data produced by those participants.^6^ We used linear mixed‐effects models with experiments and the number of encounters as fixed factors, different verbs as a random factor, and the number of unique guesses as the dependent variable. We also included an interaction term in the models. Compared to the Visual‐only condition, participants in the Visual‐CSL condition tended to have fewer unique guesses, β *=* −2.19, *SE* = 0.17, *t*(118) = −1.86, *p* = .06. But there was no significant effect of increased encounters or interaction (Encounter: β = −0.25, *SE* = 0.21, *t*(118) = −1.19, *p* = .241; Interaction: β = −0.19, *SE* = 0.30, *t*(118) = −0.62, *p* = .54). When comparing the Visual‐and‐linguistic and Visual‐and‐linguistic‐CSL conditions, there was a significant interaction between experiment and increased encounters, β = −0.93, *SE* = 0.25, *t*(118) = −3.70, *p* < .001. The Visual‐and‐linguistic‐CSL condition started with a higher number of unique guesses but gradually had fewer unique guesses than the baseline; and the differences became more pronounced in later encounters. After the interaction was taken into account, there was no significant main effect of experiment or encounter (Experiment: β = 0.59, *SE* = 0.98, *t*(118) = 0.61, *p* = .54; Encounter: β = −0.16, *SE* = 0.18, *t*(118) = −0.91, *p* = .36). These results provided further evidence that cross‐situational learning allowed the learners in Experiment 2 to reduce their guess variability.

#### 3.2.3 Convergence of guesses: The proportion of guesses belonging to the top choice

We used linear mixed‐effects models, with individual verbs as a random factor, to test the effect of increased encounters on the convergence of guesses. The proportion of responses belonging to the top choice in the Visual‐CSL condition increased across exposures, β = 0.04, *SE* = 0.01, *t*(54) = 4.24, *p* < .001 (Fig. 4b). Similar findings can be seen in the Visual‐and‐linguistic‐CSL condition, β = 0.06, *SE* = 0.01, *t*(54) = 7.23, *p* < .001. These results suggest that learners benefited from cross‐situational learning, as evidenced by higher certainty or agreement in their guesses as they extracted information across trials.

Our next set of analyses used individual verbs as a random factor and tested whether the conditions and the number of encounters affected the proportion of responses that belonged to the top choice. More guesses belonged to the top choice in the Visual‐and‐linguistic‐CSL condition than the Visual‐CSL condition, β = 0.21, *SE* = 0.07, *t*(118) = 2.90, *p* = .004. The proportions increased from early encounters to later encounters, β = 0.04, *SE* = 0.01, *t*(118) = 2.83, *p* = .005. There was no significant interaction, β = 0.03, *SE* = 0.02, *t*(118) = 1.48, *p* = .14. These results suggest that the additional linguistic information facilitated the convergence of guesses. Importantly, cross‐situational learning helped learners converge on the same top choice.

##### 3.2.3.1. Between‐experiment analyses

We next used linear mixed‐effects models with different verbs as a random factor and examined whether the proportions of responses belonging to the top choice in the two conditions of Experiment 2 were significantly higher than the baselines (i.e., Visual‐only and Visual‐and‐linguistic conditions from Experiment 1) and whether increased encounters affected the convergence of guesses. We also included an interaction term in the models. Between the Visual‐only and Visual‐CSL conditions, there was a significant interaction between experiment and encounter, β = 0.03, *SE* = 0.01, *t*(118) = 2.42, *p* = .02. The two conditions started with comparable proportions of guesses belonging to the top choice; but with increased encounters, the Visual‐CSL condition ended with higher proportions belonging to the top choice. After the interaction term was taken into account, there was no significant main effect of experiment or encounter (Experiment: β = −0.05, *SE* = 0.05, *t*(118) = −0.96, *p* = .34; Encounter: β = 0.002, *SE* = 0.01, *t*(118) = 0.26, *p* = .80). Similarly, there was a significant interaction when comparing the Visual‐and‐linguistic‐CSL condition and the baseline, with the proportions starting lower in the Visual‐and‐linguistic‐CSL condition but becoming higher with increased encounters, β = 0.05, *SE* = 0.01, *t*(118) = 3.48, *p* < .001. After the interaction term was taken into account, the main effects of experiment and encounter were not significant (Experiment: β = −0.02, *SE* = 0.06, *t*(118) = −0.27, *p* = .79; Encounter: β = 0.01, *SE* = 0.01, *t*(118) = 1.34, *p* = .18). These results suggest that participants in Experiment 2 started with similar or slightly worse performance than the baselines; but they benefited from cross‐situational learning and increased encounters facilitated the convergence of guesses.

#### 3.2.4 Accuracy: The proportion of guesses that matched the verbs produced by parents

We conducted generalized linear mixed‐effects models to test the effect of increased encounters on accuracy, with the participants, the verbs, and specific video instances as random factors. In the Visual‐CSL condition (Fig. 3c), the mean accuracy across encounters was 0.33 (*SD* = 0.47). Increased encounters with the videos of the same verb significantly increased accuracy, β = 0.25, *SE* = 0.04, *z* = 6.68, *p* < .001, *n* of observations = 2376. The mean accuracy across encounters for the Visual‐and‐linguistic‐CSL condition was 0.71 (*SD* = 0.45). The accuracy also increased as a function of increased encounters, β = 0.45, *SE* = 0.04, *z* = 11.16, *p* < .001, *n* of observations = 2376. These findings suggest that cross‐situational learning allowed participants to increase their guess accuracy.

Comparing the two conditions in Experiment 2, participants in the Visual‐and‐linguistic‐CSL condition had significantly higher accuracy than their counterparts in the Visual‐CSL condition, β = 1.33, *SE* = 0.18, *z* = 7.44, *p* < .001, *n* of observations = 4752. There was a significant effect of increased encounters, β = 0.20, *SE* = 0.03, *z* = 6.18, *p* < .001, *n* of observations = 4752. There was also a significant interaction (β = 0.18, *SE* = 0.04, *z* = 4.17, *p* < .001, *n* of observations = 4752) showing that the differences between the two conditions were larger in the later trials than in the earlier trials. This again suggested that linguistic information helped learners identify the correct verbs that parents used in the videos. More importantly, with increased encounters, the effect became more pronounced in the later trials.

##### 3.2.4.1. Between‐experiment analyses

Since the accuracy measure was calculated based on the proportion of correct guesses in each test trial, the number of participants in each group should not affect the patterns. Therefore, in the following analyses, we included all participants in Experiments 1 and 2 in the between‐experiment accuracy analyses.

We first compared the two Visual‐only conditions (Visual‐only in Experiment 1 vs. Visual‐CSL in Experiment 2) by including the main effects of the experiment and increased encounters, and an interaction term between the two factors as the predictors, and the individual participants, verbs, and video instances as random factors to predict participants’ accuracy. There was a significant interaction between experiment and increased encounters, β = 0.18, *SE* = 0.05, *z* = 3.98, *p* < .001, *n* of observations = 5346. Participants in the two conditions had similar accuracy performance in early encounters, but the accuracy became higher in the Visual‐CSL condition with increased encounters. The simple main effects of experiment and encounter were not significant after the interaction term was added to the model (Experiment: β = 0.05, *SE* = 0.22, *z* = 0.25, *p* = .81, *n* of observations = 5346; Encounter: β = 0.04, *SE* = 0.03, *z* = 1.20, *p* = .23, *n* of observations = 5346).

We then compared the two Visual‐and‐linguistic conditions (Visual‐and‐linguistic in Experiment 1 vs. Visual‐and‐linguistic‐CSL in Experiment 2). There was a significant interaction between experiment and increased encounters (β = 0.43, *SE* = 0.04, *z* = 9.97, *p* < .001, *n* of observations = 6336) and a simple main effect of increased encounters (β = 0.08, *SE* = 0.02, *z* = 3.29, *p* = .001, *n* of observations = 6336). The simple main effect of the experiment was nearly significant, β = −0.32, *SE* = 0.18, *z* = −1.79, *p* = .07, *n* of observations = 6336. As shown in Fig. 4c, the statistical analyses confirmed that the accuracy in the Visual‐and‐linguistic‐CSL condition initially started lower than the baseline but increased with more encounters, and the accuracy difference became more pronounced with increased encounters.

### 3.3 Discussion

Overall, using a blocked design allowed learners to accumulate information across different encounters. Learners were more likely to converge on their guesses and were more accurate in their guesses. In both Visual‐CSL and Visual‐and‐linguistic‐CSL conditions, the number of unique guesses decreased as a function of increased encounters. In contrast, the proportion of guesses belonging to the top choice and the accuracy increased across trials. These results indicate that learners benefited from the information aggregated across trials in their verb learning. Compared to the baselines in Experiment 1, the gain from cross‐situational learning was significant for both the Visual‐CSL and Visual‐and‐linguistic‐CSL conditions, and it became more pronounced with increased encounters.

The Sankey diagrams in Fig. 3 showed that visual information alone allowed learners to pick up some relevant (partial) knowledge regarding the actions associated with the target verbs. Additional linguistic information allowed learners to quickly narrow down the search space. Learners used accumulated information across trials to fine‐tune their guesses. Importantly, we picked a verb with the lowest overall accuracy (with an average accuracy of 0.46) in the Visual‐and‐linguistic‐CSL condition to illustrate the changes of guesses across encounters. The results showed that visual and linguistic information together can help learners converge to the correct verb in just six encounters. When considering verbs with higher average accuracies (e.g., *fall*: 0.91, *put*: 0.89, and *fit*: 0.86), the joint effects of these different sources of within‐trial information were stronger and allowed learners to reach ceiling accuracy with even fewer encounters.

## 4 General discussion

Using two experiments, we investigated how added within‐trial linguistic information helped verb learning in the contexts that either discouraged or encouraged learners to aggregate information across visual scenes. The stimuli in Experiment 1 were presented in an interleaved design, posing challenges for learners to aggregate information across trials. In this experiment, with visual information alone (i.e., Visual‐only condition), learners were able to identify some salient actions associated with the target verbs. However, likely because each action can be described using different verbs, depending on the aspect(s) that individual learners focused on (e.g., *play*, *move*, or *turn*), their guesses were highly variable. The addition of linguistic information (Visual‐and‐linguistic condition) helped learners narrow down their search space and reduced the potential verbs that can be used in a scene. The effect of linguistic information was quick and can be seen from the first encounter (Fig. 3). In Experiment 2, videos containing the same target verb were presented in a blocked manner, allowing learners to aggregate information across encounters easily. In the Visual‐CSL condition, learners used the visual information aggregated across trials to refine their guesses and eventually narrow down their guesses to a few final candidates. However, the process was more gradual and relatively slow. With the combination of visual and linguistic information across trials (i.e., Visual‐and‐linguistic‐CSL condition), the success rate was the highest. Learners were able to narrow down their search space quickly and gradually converge on a target verb. Importantly, the significant interaction effects across analyses suggested that with the accumulation of cross‐situational information, the facilitative effect of linguistic information (added on top of visual information) was enhanced and became increasingly stronger across trials.

### 4.1 Cross‐situational verb learning

Our research adds to the existing knowledge of cross‐situational word learning in several ways. First, we moved beyond word‐referent mapping and focused on word‐meaning mapping in the context of verb learning. The majority of cross‐situational word learning studies tested learning by asking learners to pick the target object or event that goes with a word, which is a word‐referent mapping problem (Akhtar & Montague, 1999; Childers & Paik, 2009; Dautriche & Chemla, 2014; Monaghan et al., 2015; Scott & Fisher, 2012; Suanda & Namy, 2012; Yu & Smith, 2007). Even though a few recent studies included designs that tapped into the word‐meaning mapping problem (e.g., whether learners map a novel word to all dogs or to a type of dog), they focused on the noun learning domain (e.g., C. Chen et al., 2018; Wang & Trueswell, 2019, 2022). Our study extends to the verb learning domain, which has long been shown to be more challenging than noun learning (Gentner, 1982; Gleitman et al., 2005; Piccin & Waxman, 2007; Snedeker & Gleitman, 2004). We demonstrated that learners utilized visual and linguistic information present within each trial to solve the word‐referent mapping problem efficiently, and importantly, cross‐situational learning enabled them to fine‐tune the word‐meaning mappings.

Second, the cross‐situational learning mechanism can operate even when there are considerable variabilities in the learning scenes. The type of stimuli used in this research is fairly different from the ones used in many previous cross‐situational learning studies (Akhtar & Montague, 1999; Benitez et al., 2016; C. Chen et al., 2018; Scott & Fisher, 2012; Suanda & Namy, 2012; Vlach & Johnson, 2013; Yu & Smith, 2007; Zettersten & Saffran, 2020). In prior studies, what learners had to do was to track cross‐situational co‐occurrences between words and objects or events and use them to learn the mappings between the two. Oftentimes, there were one‐to‐one mappings between words and their referents or meanings (for a few exceptions, see Benitez et al., 2016; C. Chen, Gershkoff‐Stowe, Wu, Cheung, & Yu, 2017, 2018; Crespo, Vlach, & Kaushanskaya, 2023; Kachergis, Yu, & Shiffrin, 2012; Poepsel & Weiss, 2014; Yurovsky et al., 2013). Therefore, what learners track or extract from the learning trials is the between‐trial regularity in the scenes. That is, they need to notice the *invariability* (or very limited variability) in the scenes, such as a specific object seen consistently across trials or several objects with similar features seen consistently across trials, and map them to corresponding words. However, in our studies, even though noticeable patterns may emerge across scenes, learners often need to track much larger variabilities across trials regarding the objects and actions involved. For example, the objects and actions associated with the verb *turn* include turning the feet of a toy that is rotatable, turning a Rubik's cube, turning the direction of a knife, and showing a claw‐like hand in a turning gesture without holding any specific object. Moreover, with some verbs, there were significant differences in the actions involved (e.g., a child using a hand to shake a toy phone, a toy fish shaking its tail, or a parent asking a child to shake an object without the action in view). From an information processing perspective, the variabilities involved in our verb learning studies are significantly larger than those seen in most previous cross‐situational learning experiments. It is also important to note that we only used concrete action verbs, which tend to be more perceptible, imaginable, and visually grounded, compared to more abstract verbs, such as mental state verbs (Gillette et al., 1999; Gleitman et al., 2005; Piccin & Waxman, 2007). The perceptual variabilities and the relatively transient nature of actions in the visual scenes may explain why visual information alone can be harder to process and seems of limited use in verb learning compared to noun learning, which often involves objects with more sustained existence in time and space. The second contribution of our research is to show that cross‐situational learning is not limited to tracking invariabilities, but can extend to learning scenes with much larger variabilities.

Third, our studies showed that learners benefited from cross‐situational learning regardless of whether they received only visual information or both visual and linguistic information in each trial. Importantly, the gain from cross‐situational learning is larger when both visual and linguistic information is available, compared to when only the visual information is available. This suggests that the effect of cross‐situational learning is enhanced when there are rich multiple sources of within‐trial information (e.g., both visual and linguistic information). Furthermore, across trials, the rich get richer at a faster rate than the poor (i.e., when only visual information is available). This same principle likely also applies to learners with different levels of linguistic knowledge, in that learners with better linguistic knowledge may be better at using linguistic cues and benefit more from rich linguistic information across different learning situations than learners with limited linguistic knowledge. Our finding expands on prior cross‐situational learning literature, which shows that learners can incorporate within‐trial information in their learning with lab‐created stimuli or well‐defined events, to the verb learning domain with naturalistic stimuli (Y. Chen et al., 2024; Dunn et al., 2024; Finley, 2023; MacDonald, Yurovsky, & Frank, 2017; Monaghan et al., 2015, 2024). Our research offers new insights into how different types of information interact in cross‐situational verb learning.

Fourth, our studies shed light on how cross‐situational word‐meaning mapping may unfold over time. One interesting observation from Fig. 4 is that the added encounters continued to help learners refine their guesses, even though the improvement appeared more noticeable in the early trials than in the later trials. This is similar to the diminishing returns phenomenon in economic theories, where the gain obtained from each unit of input increases monotonically at first, but after reaching a certain point, the gain per unit of input starts to decrease (Brue, 1993; Knight, 1944). This is probably because, after learners have gained a basic understanding of what the target verb may be in early encounters, they need more nuanced differences to distinguish between verbs with similar meanings in later encounters. For example, in the first two to three encounters, they may use the within‐trial information to identify that the target verb is about an action related to rotation. However, in later encounters, they will need more nuanced information to help them determine whether the verb is *turn* or *twist*. Depending on the nature of different types of input, learners may need a different number of encounters to achieve a good understanding of a verb. Some verbs may be acquired in a few encounters, while others may need significantly more exposure for learners to fully grasp the meaning(s).

### 4.2 Can the findings be applied to first language learning?

In the current studies, we used the HSP, in which adult participants guessed what verb a parent produced based on the information they encountered in each video (Gillette et al., 1999; Gleitman et al., 2005; Kako, 2005; Medina et al., 2011; Piccin & Waxman, 2007; Snedeker & Gleitman, 2004). The experimental conditions provide a fairly good simulation of second language learning scenarios, from having no knowledge of a language and needing to rely solely on the visual information within a scene (i.e., Visual‐only conditions) to having a fairly good grasp of a language but needing to guess the meaning of a specific verb based on all available information (i.e., Visual‐and‐linguistic‐CSL conditions). These different conditions enabled us to systematically test the separate and joint effects of visual and linguistic information on how adults infer the meaning of a mystery verb across various learning situations. However, this type of learning is different from children's first language learning in several ways.

In first language learning, children learn how adults use verbs to depict actions or relations (Merriman & Tomasello, 1995; Smiley & Huttenlocher, 1995). They hear a verb and learn that it can be used in a scene with certain characteristics or features. What children do is to learn how an action or event in a scene can be described and/or to match a verb they hear to the action or event they see. Across different situations, children use whatever information is available to enhance their understanding of the verb and gradually learn the correct usage of the verb. During the process, children may or may not consciously think about what the verb means and may only accumulate implicit (partial) knowledge about the verb (Kachergis, Yu, & Shiffrin, 2014; Yurovsky et al., 2014). On the other hand, participants in the HSP are asked to guess a verb produced by a parent based on the information provided in the video. And they need to make a guess after each exposure. This requires that they take all available information into account, consciously consider the types of verbs that can be used in the scene, and select one that they think parents are likely to use. Participants’ guesses are coded as correct or incorrect based on whether they match the exact verbs that parents produced in the videos. It has been argued that this is a very high threshold for learning (Schoener, Schoener, Johnson, & Suanda, 2023). There are many reasons why a guess could go wrong. First, the participant may notice an action that differs from what the parent referred to in the scene (e.g., *reach* vs. *turn*). Second, they can pick the same action but use a different verb to describe it (e.g., *turn* vs. *twist*, Zhang, Amatuni, Crain, & Yu, 2020). Third, theoretically, they can choose the same action but focus on a different perspective (e.g., *give* vs. *receive* or *chase* vs. *flee*; Gleitman et al., 2005), even though this explanation may not apply to our stimuli. Another important difference between adults’ learning in the current studies and children's first language learning is that adults have better cognitive and linguistic abilities and may process information differently than young children (e.g., Benitez, Zettersten, & Wojcik, 2020; Bloom & Kelemen, 1995; Cerella & Hale, 1994; Frankel & Howard, 1985; Kail, 2000; Piccin & Waxman, 2007). Some studies using explicit measures (e.g., explicit pointing) suggest that young children struggle and often fail to track information cross‐situationally (Aravind et al., 2018; Wang, Luo, & Li, 2024; Wang & Trueswell, 2019; Woodard, Gleitman, & Trueswell, 2016). However, another line of research, which often used implicit measures (e.g., looking time or error analyses), showed that young children and even infants could track more than one‐to‐one word‐object mappings and often contextual information cross‐situationally (C. Chen & Yu, 2022; Knabe & Vlach, 2023; Suanda et al., 2014; Vlach & Sandhofer, 2011; Vouloumanos & Werker, 2009). The inconsistent findings in the literature are likely because one line of research tapped on explicit knowledge, while the other tapped on implicit knowledge. Recently, Sia and Mayor (2021) employed explicit pointing measures to examine the cross‐situational word learning ability of 4‐ to 12‐year‐olds. They found that children over 6 years of age tracked both the mappings between novel words and novel objects and the associations between the novel words and their co‐occurring familiar objects cross‐situationally. Importantly, children's ability to track multiple associations increases with age. Nonetheless, the different conclusions drawn from these studies highlight the need for further developmental research to determine whether our findings can be directly applied to children's learning.

Although our designs differ from children's daily word learning, the results of our research still demonstrate the in‐principle utility of different information sources in language learning and how learners may progress as they become more capable of utilizing various types of information (Kako, 2005). When an infant or a young child has no or minimal linguistic knowledge, the primary cue they rely on is likely the salient visual information. Early in development, visual information likely plays a much more critical role than linguistic information (Kako, 2005). As seen from the Visual‐only condition, visual information alone is often insufficient for successful verb learning. Learners may gather some relevant information based on a visual scene and accumulate partial knowledge about the event they observe. After accumulating partial knowledge across several encounters, many likely reach a good enough understanding of a verb and can reasonably guess the verb's meaning, as seen in the Visual‐CSL condition. However, the progress made on this route is likely to be very slow and often not precise enough. On the other hand, with the help of linguistic information (Visual‐and‐linguistic condition), learners can quickly narrow down the search space. After children acquire more linguistic knowledge, they may begin to apply it in their learning of novel verbs (Messenger, Yuan, & Fisher, 2015; Scott & Fisher, 2012; Yuan & Fisher, 2009). A recent study showed that novel verbs attract preschoolers’ attention to actions, rather than actors of the actions, and help them zoom in on the key elements that are critical for verb learning (Childers et al., 2022). However, without accumulating information across situations, the effect of linguistic information can be relatively limited, as seen in the Visual‐and‐linguistic condition. With the ability to accumulate information across different situations, the visual and linguistic information together allows learners to quickly narrow down their search at an early stage of learning and then gradually fine‐tune their learning (Visual‐and‐linguistic‐CSL condition). Therefore, each type of information plays a different, but complementary, or even facilitative, role in cross‐situational verb learning. The general learning principles regarding how different types of cues affect within‐trial information selection and between‐trial information integration are not restricted to first or second language learning, but can be extended to more general learning domains, such as pattern or rule extraction (Gentner, 1983; Marcus et al., 2007), structure or sequence learning (Baker et al., 2004; Turk‐Browne et al., 2005; Turk‐Browne & Scholl, 2009), and forming associations between events and objects (Wasserman & Miller, 1997).

It has been argued that linguistic information can help learners discern transitive versus intransitive verbs, as transitive verbs usually appear with two nouns (e.g., The boy pushed the girl), while intransitive verbs usually appear with one noun (e.g., The boy jumped; Messenger et al., 2015; Yuan & Fisher, 2009). However, in real‐life child‐directed speech, the utterances often do not contain linguistic structures in the “perfect forms.” For example, utterances often contain directives or incomplete sentences in which the actor of an action can only be inferred from the scene (e.g., “Here, hold here.” “Shake it.” “Drive the train.”). This may be one of the reasons why learners’ overall accuracy was still less than perfect even in the Visual‐and‐linguistic‐CSL condition in our study. However, it is worth noting that, despite having less‐than‐perfect performance, learners still achieved high accuracy in the final few trials (i.e., over 80% accuracy). This suggests that cross‐situational learning allows learners to comprehensively understand how a verb may be used. Importantly, in our studies, there were only six encounters per verb. In real life, children often hear verbs much more frequently, with dozens to even hundreds of exposures (Snedeker & Gleitman, 2004). Therefore, even though the linguistic information in a specific utterance may not be ideal or even beneficial, the linguistic information accumulated through more cross‐situational exposures should enable learners to understand different words effectively.

Another relevant issue is that in Experiment 2, which investigated the role of cross‐situational learning in the verb learning domain, the trials were presented in a blocked manner. It has been argued that, in daily life, children are likely to be exposed to new words “sporadically rather than in uninterrupted succession” (Gillette et al., 1999). However, recent studies analyzing children's language and visual input from daily interactions reveal two important characteristics: burstiness and a tendency to return to frequently discussed topics (Karmazyn‐Raz & Smith, 2023; Slone, Abney, Smith, & Yu, 2023). When parents interact with their children, they tend to make repeated references to the same object in close proximity and revisit the same topics over time (Suanda et al., 2016). Similarly, they tend to organize their interactions around the same actions (e.g., shaking a rattle and then shaking a ball or stacking blocks and then stacking chairs) and talk about the actions at the same time (Karmazyn‐Raz & Smith, 2023). During the interactions, they switch between activities and conversation topics. However, over time, they tend to return to the frequent activities and topics repeatedly. The clustered use of a word in close succession is similar to presenting words in a blocked design, while the return to frequent topics is similar to presenting words in an interleaved or interspersed design. Therefore, both blocked and interleaved stimulus presentations capture certain aspects of children's daily language and visual input and are not mutually exclusive from each other.

### 4.3 Can the findings be applied to hard(er) verbs or verbs with similar meanings?

The verbs we used in the current studies were all concrete action verbs, which tended to be perceptible, imaginable, and visually grounded (Gillette et al., 1999; Gleitman et al., 2005; Piccin & Waxman, 2007). One question to ask is whether the findings can be applied to verbs that are more abstract, such as mental state verbs, *think*, *miss*, or *love*. It has been argued that linguistic information plays a more important role than visual information when learning abstract verbs (Gleitman et al., 2005; Kako, 2005; Piccin & Waxman, 2007). One reason is that the observable actions associated with abstract verbs may be a lot more variable; and, thus, this makes it harder to extract specific patterns from the visual scenes (e.g., seeing someone frowning while thinking, seeing another person looking up to the ceiling while thinking, yet a different person closing eyes while thinking). On the other hand, it has also been argued that, to learn the content meaning of a verb successfully, observations of the external contexts (e.g., hearing a word being used in a context) and/or internal states (e.g., observing one's own thinking and belief) are important (C. Fisher et al., 2010; Pinker, 1994). Very young children sometimes pick up the salient actions associated with a mental state verb, for example, hugging and kissing for the verb *love* (Piccin & Waxman, 2007). These “acts of love” do teach them something about *love*. Taken together, even though visual information in one scene may not be particularly useful for learning abstract verbs, visual and linguistic information accumulated across different situations should still be beneficial, as they allow learners to enhance their understanding of verb meanings.

Another question to ask is how children learn verbs with similar meanings, such as *turn* and *twist*. One possible solution is to increase the number of encounters and the variability of visual and linguistic information. Most of the videos we used for the verb *turn* involved someone turning part of an object (e.g., the feet of a rotatable toy or a Rubik's cube). In those videos, the actions can often be described using the verb *twist* as well. Exposure to more variable visual and linguistic contextual information will enable learners to discern the nuanced differences between verbs (e.g., turning a key vs. twisting a key; or a car turns left vs. a person twists a towel). With more variable learning situations or contexts, learners can use various sources of accumulated information to discover the whole meaning space associated with a specific verb. Learning hard verbs or verbs with similar meanings using this method likely requires a lot of exposure and can take a long time. Our research provides evidence that some verbs can be learned within a few encounters. But young children have a different organization in their early semantic space than adults (A. V. Fisher, Godwin, Matlen, & Unger, 2015; Unger, Fisher, Nugent, Ventura, & MacLellan, 2016). They keep developing and updating their knowledge as new information comes in. With intensive exposure, learning‐related changes can be seen in a few days (Unger et al., 2016). But many changes often take much longer—on a timescale of months or years. A second method of learning is much faster and can be achieved by using social cues or pedagogical explanations (Bonawitz et al., 2011; Csibra & Gergely, 2009; Frank, Tenenbaum, & Fernald, 2013; Shafto, Goodman, & Frank, 2012). Parents (and other people) usually transmit knowledge to children in daily interactions. This provides a “short‐cut” for children to learn how different words can be used. These different pathways may all lead to successful learning of verbs with similar meanings.

## 5 Conclusions

We examined how the addition of linguistic information aided cross‐situational verb learning using visual scenes extracted from naturalistic parent−child play. We found that, even though visual information alone allowed learners to accumulate partial knowledge of a verb's meaning, it is usually not sufficient for a comprehensive understanding of the verb's meaning. Linguistic information enabled learners to quickly narrow down the search space and focus on a few relevant aspects. Cross‐situational learning allowed learners to fine‐tune their understanding. Learners used in‐the‐moment cues aggregated across learning trials to quickly identify the relevant information necessary to solve the word‐referent mapping problem and gradually improve their understanding of a verb to solve the word‐meaning mapping problem. The findings provide important insights into both first and second language learning.

## Conflict of interest statement

The authors declare no conflict of interest.

## Data Availability

The data and analyses can be found on the Open Science Framework (OSF) site [https://osf.io/b7mjz/?view_only=1d06291e5f814016804525175a187559].

## Notes

1 Notionally, we use capital letters to represent concepts, italics for words, and double quotes for utterances.

2 According to the WordBank norms based on the MacArthur‐Bates Communicative Development Inventories (Frank, Braginsky, Yurovsky, & Marchman, 2017), the top five verbs that were understood by infants by 12 months of age were all concrete action verbs: eat (57% of 12‐month‐olds), kiss (57%), drink (45%), hug (42%), and give (38%).

3 Previous studies using the HSP suggested that between 12 and 20 participants per condition would be sufficient to detect significant accuracy differences when learners received visual‐only compared to visual‐and‐linguistic information (Gillette, Gleitman, Gleitman, & Lederer, 1999, Experiment 3; Piccin & Waxman, 2007; Gleitman, Cassidy, Nappa, Papafragou, & Trueswell, 2005). However, since our studies included three different measures, variability, convergence, and accuracy of guesses, we decided to use a larger sample size than those studies. Inspired by the sample sizes in Medina, Snedeker, Trueswell, and Gleitman (2011), we aimed to recruit a minimum of 36 participants and a maximum of 60. Due to the Covid‐19 pandemic, our in‐lab experiments had to stop when we had recruited 52 participants in the Visual‐only condition. When we recruited online participants for the Visual‐and‐Linguistic condition, we reached the goal of getting 60 participants in the final sample size.

4 For the verbs we used in the studies, on average, each verb contained videos coming from 5.55 dyads (range: 5−6). That is, for most of the verbs, each video came from a different dyad from the corpus. And a dyad never contributed more than two videos per verb.

5 A few utterances started with “Oh” or “Ah” and followed by the target verb (e.g., “Oh, knock down!”). Syntactically, we consider these utterances having the verbs at the utterance‐initial positions.

6 We also calculated the numbers of unique guesses based on the data produced by two other subsets of participants with one using 36 randomly selected participants and the other using the last 36 participants in each condition. The overall conclusions were the same.
